# Differential diagnosis of frontotemporal dementia subtypes with explainable deep learning on structural MRI

**DOI:** 10.3389/fnins.2024.1331677

**Published:** 2024-02-07

**Authors:** Da Ma, Jane Stocks, Howard Rosen, Kejal Kantarci, Samuel N. Lockhart, James R. Bateman, Suzanne Craft, Metin N. Gurcan, Karteek Popuri, Mirza Faisal Beg, Lei Wang, on behalf of the ALLFTD consortium

**Affiliations:** ^1^Department of Internal Medicine, Wake Forest University School of Medicine, Winston-Salem, NC, United States; ^2^Department of Psychiatry and Behavioral Health, Northwestern University Feinberg School of Medicine, Chicago, IL, United States; ^3^Weill Institute for Neurosciences, University of California San Francisco, San Francisco, CA, United States; ^4^Department of Radiology, Mayo Clinic, Rochester, MN, United States; ^5^Department of Neurology, Wake Forest University School of Medicine, Winston-Salem, NC, United States; ^6^Department of Computer Science, Memorial University of Newfoundland, St. John's, NL, Canada; ^7^School of Engineering Science, Simon Fraser University, Burnaby, BC, Canada; ^8^Department of Psychiatry and Behavioral Health, Ohio State University Wexner Medical Center, Columbus, OH, United States

**Keywords:** FTD (frontotemporal dementia), differential diagnosis algorithm, explainable deep learning, multi-type features, multi-level feature fusion, bvFTD, nfvPPA, svPPA

## Abstract

**Background:**

Frontotemporal dementia (FTD) represents a collection of neurobehavioral and neurocognitive syndromes that are associated with a significant degree of clinical, pathological, and genetic heterogeneity. Such heterogeneity hinders the identification of effective biomarkers, preventing effective targeted recruitment of participants in clinical trials for developing potential interventions and treatments. In the present study, we aim to automatically differentiate patients with three clinical phenotypes of FTD, behavioral-variant FTD (bvFTD), semantic variant PPA (svPPA), and nonfluent variant PPA (nfvPPA), based on their structural MRI by training a deep neural network (DNN).

**Methods:**

Data from 277 FTD patients (173 bvFTD, 63 nfvPPA, and 41 svPPA) recruited from two multi-site neuroimaging datasets: the Frontotemporal Lobar Degeneration Neuroimaging Initiative and the ARTFL-LEFFTDS Longitudinal Frontotemporal Lobar Degeneration databases. Raw T1-weighted MRI data were preprocessed and parcellated into patch-based ROIs, with cortical thickness and volume features extracted and harmonized to control the confounding effects of sex, age, total intracranial volume, cohort, and scanner difference. A multi-type parallel feature embedding framework was trained to classify three FTD subtypes with a weighted cross-entropy loss function used to account for unbalanced sample sizes. Feature visualization was achieved through post-hoc analysis using an integrated gradient approach.

**Results:**

The proposed differential diagnosis framework achieved a mean balanced accuracy of 0.80 for bvFTD, 0.82 for nfvPPA, 0.89 for svPPA, and an overall balanced accuracy of 0.84. Feature importance maps showed more localized differential patterns among different FTD subtypes compared to groupwise statistical mapping.

**Conclusion:**

In this study, we demonstrated the efficiency and effectiveness of using explainable deep-learning-based parallel feature embedding and visualization framework on MRI-derived multi-type structural patterns to differentiate three clinically defined subphenotypes of FTD: bvFTD, nfvPPA, and svPPA, which could help with the identification of at-risk populations for early and precise diagnosis for intervention planning.

## 1 Introduction

Frontotemporal dementia (FTD) is an umbrella term describing the many clinical syndromes underlain by frontotemporal lobar degeneration (FTLD) neuropathology. FTD is characterized by the progressive impairment of cognitive and behavioral functions such as executive functioning, language, social comportment, and motor functioning (Dickerson and Atri, 2014). FTLD is the third most common cause of dementia and is as common as Alzheimer’s disease (AD) in individuals under the age of 65 (Erkkinen et al., 2018). Clinically, FTLD is typically associated with one of several diagnoses characterized by specific constellations of symptoms. Patients who present with early impairments in social comportment and executive dysfunction are typically diagnosed with behavioral-variant FTD (bvFTD). Primary progressive aphasia (PPA) is a clinical syndrome characterized by a selective deterioration of language functions and can be further subdivided into semantic (svPPA) and nonfluent variants (nfvPPA) (Mesulam et al., 2014). Regardless of the initial clinical syndrome, FTD syndromes eventually result in global dementia and death (Mioshi et al., 2010).

Although clinical trials of potential disease-altering therapies (e.g., anti-tau antibodies, tau aggregation inhibitors) are currently underway (Boxer et al., 2013; Tsai and Boxer, 2016; Mis et al., 2017; Logroscino et al., 2019; Panza et al., 2020; Huang et al., 2023), the significant degree of clinical, pathological and genetic heterogeneity observed in FTD hinders the development of sensitive and specific biomarkers that would allow for targeted recruitment of groups at highest risk for clinical/cognitive decline (Katzeff et al., 2022). Critically, early and accurate diagnosis of the clinical syndrome is essential for the targeted recruitment of participants in clinical trials, as treatments will only be effective if patients are accurately diagnosed. In bvFTD, patients show significant gray matter volume loss of the frontal and temporal lobes, with early and most distinctive loss of volume in the insula and anterior cingulate cortex (Seeley et al., 2008; Mandelli et al., 2016; Ranasinghe et al., 2016). Among the PPA syndromes, svPPA is associated with striking asymmetric (typically left > right) atrophy of the temporal pole, while nfvPPA shows atrophy of the left inferior frontal/insular cortex (Agosta et al., 2015). Across FTD clinical phenotypes, the spatial distribution of atrophy is consistent with the constellation of clinical symptoms.

While each FTD clinical syndrome has a typical anatomical pattern of neurodegeneration, early manifestations can vary greatly across people. Moreover, early patterns of neurodegeneration can be highly overlapping across clinical syndromes, such as in the case of anterior temporal lobe atrophy for both svPPA and bvFTD, and inferior frontal and insular atrophy in both bvFTD and nfvPPA. Indeed, Vijverberg et al. (2016) found that a visual review of a single MRI had insufficient sensitivity (70%) to identify cases with bvFTD. Researchers have therefore attempted to employ machine learning methods for pattern analysis to improve the classification and diagnosis of FTD (Ducharme, 2023). Similar research in the field of AD has achieved high accuracy levels when classifying diseased individuals compared to controls (often >90% accuracy) (Falahati et al., 2014; Rathore et al., 2017). Similarly, several studies have demonstrated that machine learning methods can aid in the reliable discrimination of AD and FTD (Ma et al., 2020, 2021). However, the use of machine learning methods for discrimination between FTD syndromes is rarer (see McCarthy et al., 2018 for review), often only covering a few subtypes (Wilson et al., 2009; Bisenius et al., 2017; Di Benedetto et al., 2022). Both Wilson et al. (2009) and Bisenius et al. (2017) classified PPA subtypes against each other using a principal component analysis approach based on gray matter volume, particularly for the comparison of svPPA from nfvPPA, finding moderately high accuracy (89.1%), sensitivity (84.44%) and specificity (93.8%), equivalent to an balanced accuracy of 89.1%. Similarly, Kim et al. (2019) classified bvFTD, nfvPPA and svPPA using principal component analysis and hierarchical classification and reached moderately high accuracy (overall balanced accuracy of 79.9% with 67.1% sensitivity and 92.6% specificity, and lower specificity when comparison between each FTD subtypes). Di Benedetto et al. (2022) compared different deep learning approaches but specifically for detecting bvFTD population only, and reported balanced accuracy ranging from 73.6 to 91.0% through independent validation.

In the present study, we trained a deep neural network classifier to differentiate bvFTD, nfvPPA, and svPPA patients using a multi-level feature embedding and fusion framework on multi-type morphological features derived from T1-weighted MRI scans drawn from two multi-site neuroimaging consortiums. To our knowledge, this is the first study using deep learning to examine the multi-class discrimination of all three FTD subtypes (bvFTD, nfvPPA, and svPPA) using multi-type MRI-based features.

## 2 Materials and methods

**Figure 1:**
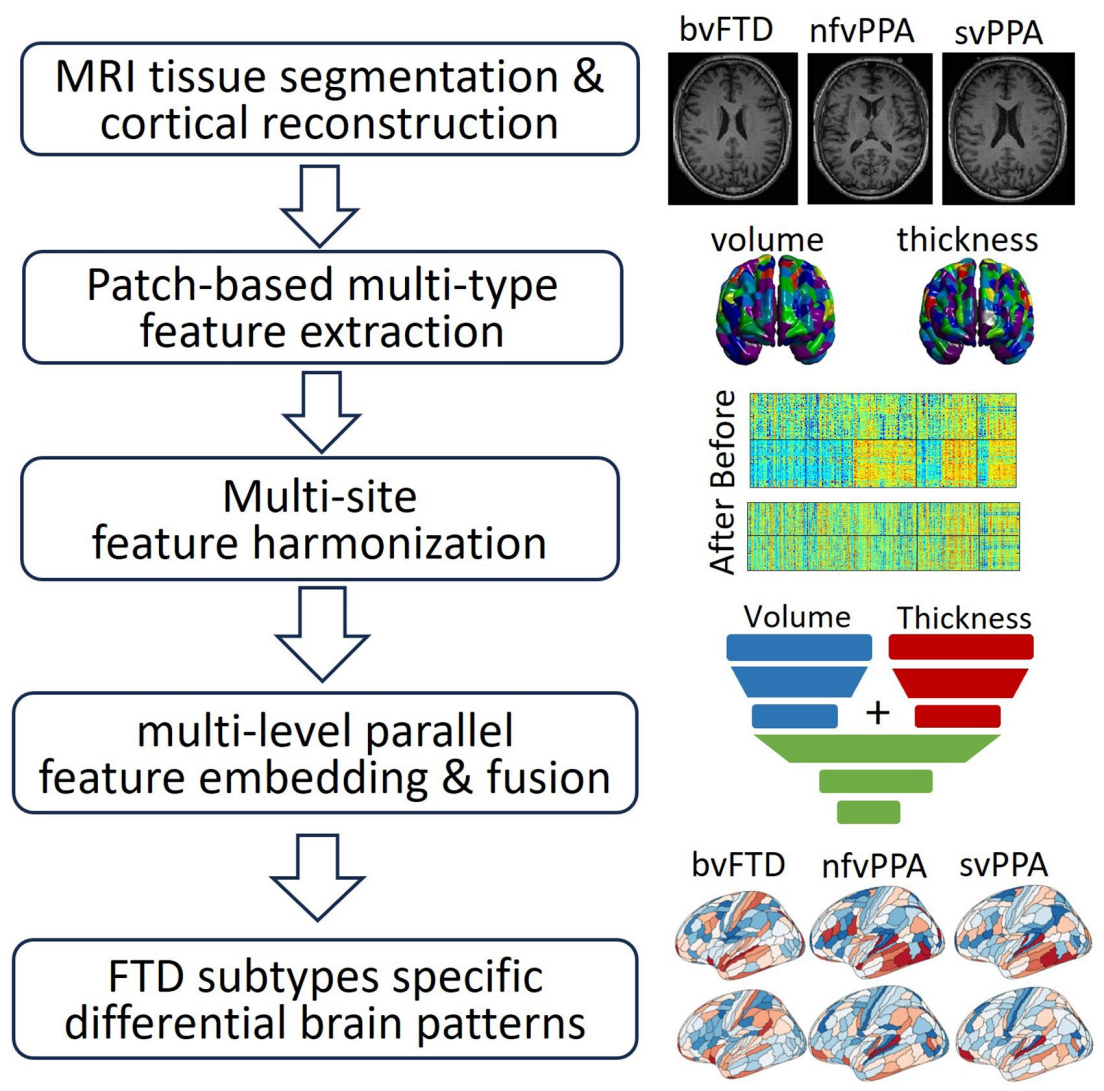
Schematic diagram of the framework in this study, which consists of: (1) feature extraction to derive patch-based multi-type features of cortical thickness and cortical/subcortical volumes; (2) W-score-based feature harmonization to control confounding factors such as scanner difference and study site bias, as well as demographic-related covariates; (3) the differential diagnosis model using multi-layer-perceptron (MLP)-based multi-level parallel feature embedding deep neural network to achieve FTD subtype classification; (4) neuroimaging-derived brain differential patterns among different FTD subtypes.

The overall schematic diagram of the proposed neuroimaging-based differential diagnosis framework is shown in Figure 1. The framework consists of four major steps: (1) feature extraction to derive patch-based multi-type features of cortical thickness and cortical/subcortical volumes; (2) W-score-based feature harmonization to control confounding factors such as scanner difference and study site bias, as well as demographic-related covariates; (3) the differential diagnosis model using multi-layer-perceptron (MLP)-based multi-level parallel feature embedding deep neural network to achieve FTD subtype classification; and (4) neuroimaging-derived feature visualization that differentiates FTD subtypes.

### 2.1 Experimental data

The experimental data consists of 173 bvFTD patients, 63 nfvPPA patients, and 41 svPPA patients, aggregated from the baseline visit studies of two cohorts: the ARTFL-LEFFTDS Longitudinal Frontotemporal Lobar Degeneration (ALLFTD) cohort (Rosen et al., 2020) and the Frontotemporal Lobar Degeneration Neuroimaging Initiative (FTLDNI, also referred to as NIFD) cohort (Boeve et al., 2019). We excluded the cognitively normal healthy subjects in the aggregated dataset due to the limited sample size (*n* = 27). Table 1 shows patient demographic information. The clinical diagnosis of FTD subtypes was defined as the ground truth to train the proposed differential diagnosis framework, regardless of their mutation carrier status.

**Table 1:** Demographics information of the patients collected from multiple cohorts, in terms of sample size and age, stratified by sex, study cohort, as well as FTD subtypes.

		Overall	Grouped by Sex	
			Male	Female
Sample size (%)		277	151 (54.5%)	126 (45.5%)
Age, mean (SD)		63.7 (7.7)	63.5 (6.9)	63.9 (8.6)
Cohort, n (%)	ALLFTD	131 (47.3%)	79 (52.3%)	52 (41.3%)
	NIFD	146 (52.7%)	72 (47.7%)	74 (58.7%)
Subtype, n (%)	bvFTD	173 (62.5%)	97 (64.2%)	76 (60.3%)
	nfvPPA	63 (22.7%)	32 (21.2%)	31 (24.6%)
	svPPA	41 (14.8%)	22 (14.6%)	19 (15.1%)

ALLFTD is a multi-site study consisting of data collected from 23 North American institutions, which is a combination of two previously independently initiated longitudinal neuroimaging studies, ARTFL and LEFFTDS. It aims to longitudinally follow FTLD mutation carriers to improve understanding of the FTLD disease progression based on both biological markers and clinical manifestation. Participants were primarily enrolled based on probable familial FTLD due to family history (i.e., with prior enrollment of a symptomatic proband), along with a small percentage of symptomatic and asymptomatic non-carriers enrolled. Mutation carriers of *MAPT*, *GRN*, or *C9orf72* genes were most common. Clinical consensus diagnosis for each clinical subtype was conducted by multidisciplinary teams following widely accepted published criteria (Gorno-Tempini et al., 2011; Rascovsky et al., 2011) and included comprehensive neurologic assessment, neuropsychological testing, brain MRI, and biofluid collection, as well an interview with caregiver or companion. Detailed information regarding the subject recruitment, diagnostic criteria, neuroimaging scanning protocols as well as image processing are available at ^1^^,^^2^.

NIFD is also a multi-site cohort with both clinical and MRI data collected at the University of California San Francisco, Mayo Clinic Rochester, and Massachusetts General Hospital. The NIFD consortium was initiated in 2010. NIFD did not collect information regarding familial mutations, and the comprehensive clinical evaluation for consensus diagnoses of FTD subtypes follows the similar criteria of ALLFTD, which includes neurologic history, neuropsychological testing, neurologic and physical examinations, structured interviews with caregiver, and neuroimaging. Detailed information regarding the subject recruitment, diagnostic criteria, neuroimaging scanning protocols, and image processing are available at ^3^.

### 2.2 Image preprocessing and patch-based multi-level multi-type feature extraction

#### 2.2.1 Brain anatomical structural parcellation and patch segmentation

Deep learning approaches such as convolutional neural network (CNN) require large-sample data to train. However, our sample size does not lend itself to those methods. Therefore, we designed a multi-type feature extraction and multi-level feature embedding framework based on a multi-layer perceptron (MLP) architecture that is appropriate for this sample size. We employed neuroimaging-based preprocessing pipelines to extract the structural features from the raw T1 MR. Two primary structural feature types were extracted from the raw T1 structural MRI data: the regional brain structure volume and cortical mantle thickness. Each MRI scan was parcellated into small patch-based features (also called super-pixels) to reduce the dimensionality of the input data while preserving anatomically relevant MRI features.

The manifold of cerebral cortical surface data was first derived through brain tissue segmentation (gray matter, white matter, and cerebral spinal fluid – CSF), followed by cortical surface reconstruction using FreeSurfer 5.3 (Fischl, 2012). The initial vertex-based data was then further segmented into 360 patches, or regions of interest (ROIs), using the HCP-MMP1 atlas (Glasser et al., 2016) to preserve critical local discriminative features. The mean cortical measurements, both volume and thickness, were then calculated for each patch as the input features. In addition, the volumes of 15 FreeSurfer-segmented subcortical gray matter structures were also included as additional volumetric features (thalamus, caudate, putamen, pallidum, hippocampus, amygdala, accumbent, both left and right hemisphere, plus brainstem). The final multi-type features resulted in a total of 735 features: 360 cortical thickness features plus 360 cortical volume features, as well as 15 subcortical volume features.

#### 2.2.2 Feature harmonization

When combining multi-cohort data, confounding factors such as demographic variation as well as discrepancies within the data acquisition devices and protocols will introduce unwanted heterogeneity within the data. Such data heterogeneity not only reduces the power of the analysis but may also introduce systematic bias. Neuroimage-derived measurements such as cortical thickness and subcortical volume will likely inherit such confounder-induced intrinsic biases. To control the confounders including cohort difference, scanner and coil difference, sex, as well as total intracranial volume (TIV), we used the generalized linear model (GLM)-based data harmonization that we have previously developed (Ma et al., 2019), using bvFTD as the reference group to calculate the reference mean and standard deviation. The resulting standard-residual term of the original feature, which is termed as w-score, is then used as the harmonized feature for the downstream tasks. It is worth noting that the GLM model used for feature harmonization was constructed using only the training data in each validation fold in the cross-validation. Details about cross-validation are described in the “model training and evaluation” section below.

### 2.3 Deep neural network (DNN)-based FTD subtype differential diagnosis model

#### 2.3.1 Neural network architecture design

**Figure 2:**
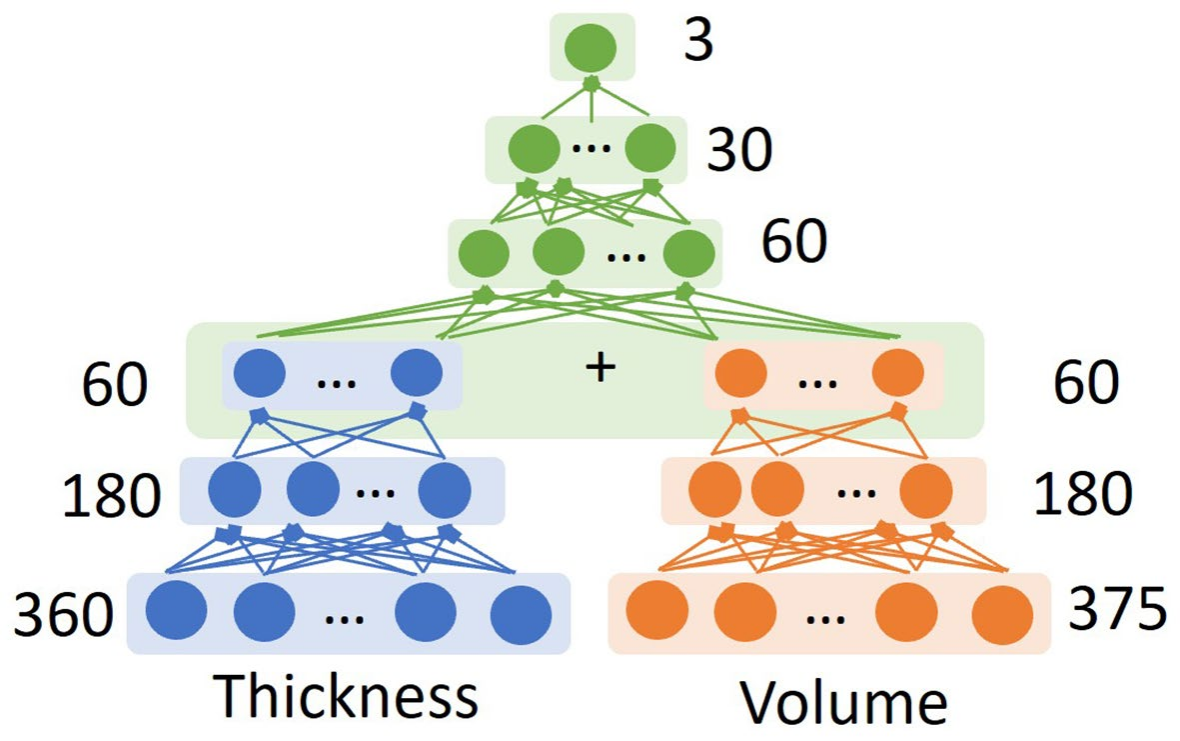
The schematic diagram of the neural network architecture of the multi-level parallel feature embedding framework used in this study to achieve accurate classification to differentiate FTD subtypes. Each block represents a multi-layer-perceptron (MLP) block. The number displayed in each of the MLP-based feature embedding blocks indicates the number of nodes in the corresponding layer.

To achieve accurate differentiation between the three FTD subtypes based on neuroimaging information, we designed and trained a deep neural network (DNN) classifier through a two-level multi-type parallel feature embedding and fusion process (Figure 2). Each of the feature-embedding blocks was built using a multi-layer perceptron (MLP). Specifically, both the patch-wise cortical thickness features and cortical/subcortical volume features were fed into the two parallel input arms of the first-level network (shown in blue and red blocks) and optimized simultaneously. The embedded features from the first level were then concatenated into a fused intermediate latent feature vector and fed into the second level network (shown in green blocks), to derive the final output node of three classes of FTD subtypes.

#### 2.3.2 Model training

A 10-fold nested cross-validation procedure was used to evaluate the robustness of the classification model, with each fold containing 80% training data, 10% validation data, and the remaining 10% of the data reserved as the independent testing set. The train/validation/test split was stratified based on the sample size ratio among FTD subtypes to ensure a comparable percentage sample for each class in each fold. The final predicted subtype classifications were derived from the probabilistic ensemble of the nine models trained in the inner folds. Weighted cross-entropy loss function was used to account for unbalanced sample size across subtypes, with weights calculated as the inverse proportion of class samples for each class. Stochastic gradient descent was used to optimize the model parameters of the DNN to minimize the loss function, with a learning rate of 1 × 10^−3^ and an L2 weight decay rate of 1 × 10^−5^.

#### 2.3.3 Performance evaluation and ablation study

To evaluate the classification performance of the differential diagnosis model, we measured the balanced accuracy for each FTD subtype, which was defined as the mean of sensitivity (the true positive rate) and specificity (the true negative rate), as well as the overall balanced accuracy calculated as the averaged across all FTD subtypes. We performed model comparisons to evaluate the effect of each component of the multi-type, multi-level feature embedding framework. A set of different experimental setups were included: (1) the proposed multi-level multi-type parallel feature embedding framework, in which the volume and thickness features were embedded into latent feature space independently in the first level before fusing and feeding into the second-level feature embedding block; (2) “naïve concatenation” model that concatenated the volume and thickness input features into a long feature vector as a naïvely-fused multi-type feature and trained a conventional MLP network with the same number of nodes at each level; (3) ablation model that used only the thickness features as input; and (4) ablation model that only used volume features as input. All the model evaluations were performed on the test sets across all 10 outer folds.

### 2.4 Clinical explainability via local feature importance

To investigate the local distinguishable structural features that contribute more toward differentiating FTD subtypes, we used an explainable AI (XAI) approach called “Integrated Gradient” (Sundararajan et al., 2017), which assigned importance scores to each input feature (i.e., volume and thickness patches) reflecting their relevant contribution to the model’s outcome prediction. This was achieved by computing the integral of the gradients of the predicted output for the given input features. The populational mean Integrated Gradient based feature importance map of each FTD subtype was then projected onto the template cortical manifold (HCP-MMP1 atlas) using the R package *ggseg* (Mowinckel and Vidal-Piñeiro, 2020). Additionally, for both volume and thickness, we conducted patch-wise linear models with the diagnostic group (vs. other groups) as the main effect and age, sex, and education as covariates. Multiple comparisons for the patch-wise cortical statistical mapping was controlled with a false discovery rate (FDR) set to 0.05.

## 3 Results

### 3.1 Multi-type structural feature extraction and harmonization

**Figure 3:**
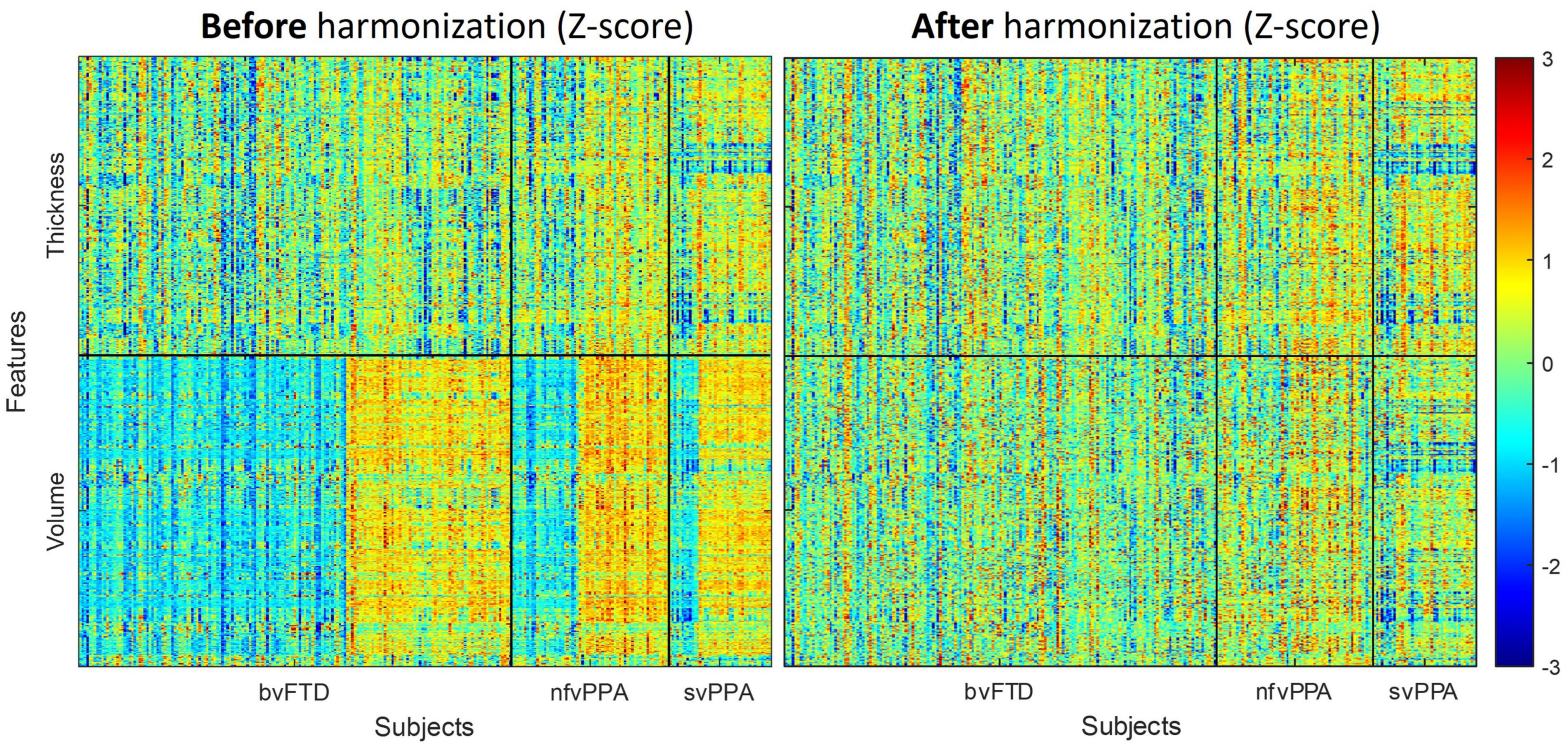
Effects of feature harmonization in preprocessings. The paranomic heatmap shows the Z-scores of thickness and volume features before (left) and after (right) the data harmonization. Z-score values for each features represents the difference between individual measurements compared to the reference group mean, standardized by the reference group standard deviation. Negative Z-scores indicate lower value than the reference mean (i.e., smaller volume, thinner cortex), while positive Z-scores represent higher value than reference mean (i.e., larger volume, thicker cortex). Cohort-dependent biases were noticeable before the harmonization (left), which were reduced after the GLM-based feature harmonization step (right).

Figure 3 displays the panorama visualization of the Z-scores for each of the input features (columns) across the entire sample population of patients (rows) for all three FTD subtypes, both before and after the feature harmonization. Z-score value for each feature represents the difference between individual measurements compared to the reference group mean, standardized by the reference group standard deviation. Negative Z-scores indicate values lower than the reference mean (i.e., smaller volume, thinner cortex); while positive Z-scores represent higher than the reference mean (i.e., larger volume, thicker cortex). The raw volumetric features showed a significant cohort effect between the ALLFTD and NIFTD data compared to the thickness feature (Figure 3 left). Comparatively, no visible cohort bias was observable after the feature harmonization (Figure 3 right).

### 3.2 Differential diagnosis model evaluation and ablation study

**Figure 4:**
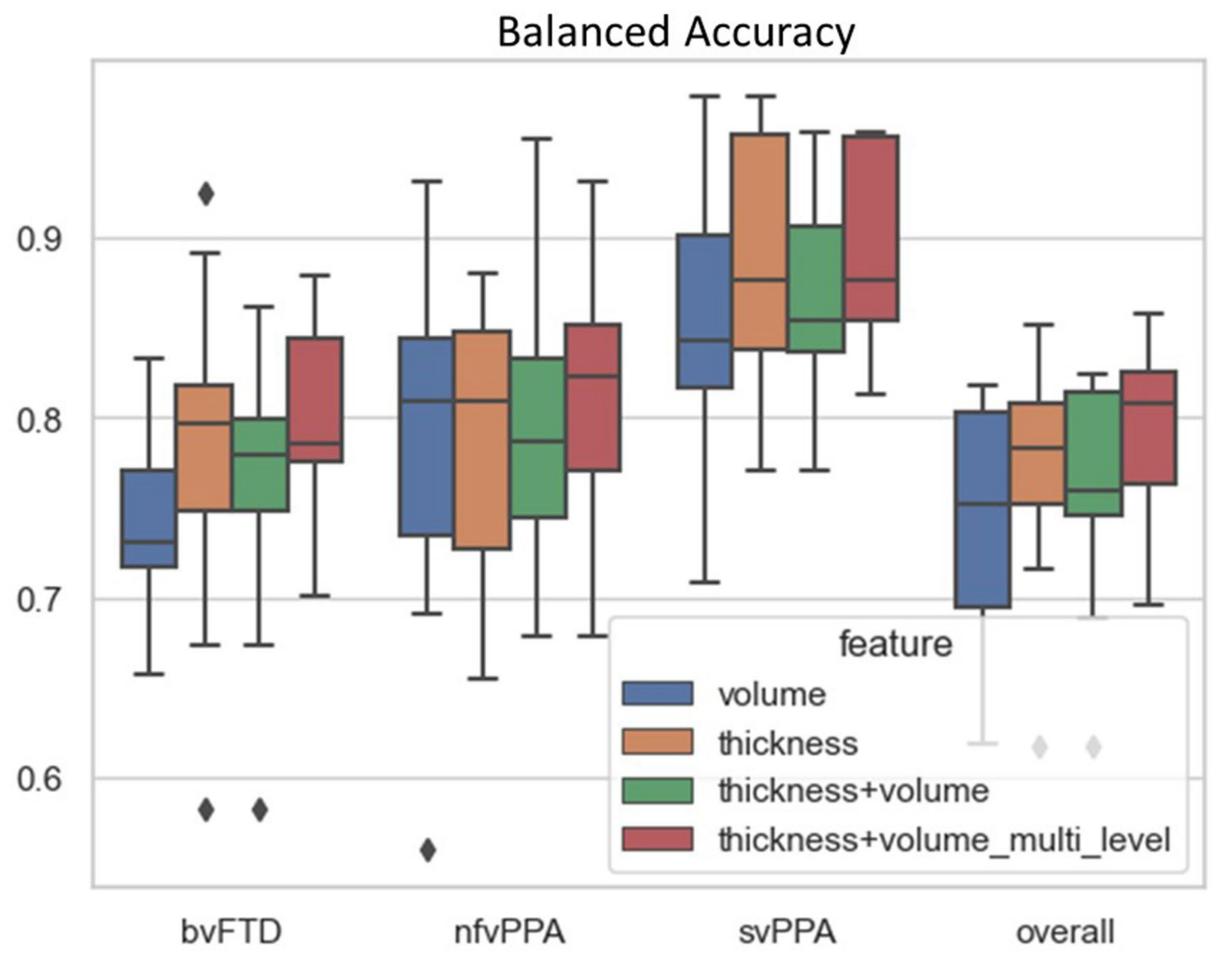
Classification performance in terms of balanced accuracy comparison among different combination of input features and network architecture design: (1) volume-only input features; (2) thickness-only input features; (3) Joint volume and thickness input feature using single-level MLP; and (4) Joint volume and thickness input feature using two-level MLP. The balanced accuracy for each individual FTD subtype (bvFTD, nfvPPA, and svPPA) as well as overall balance accuracy was reported. The black diamond box both in each subtype group as well as the overall performance represent the values of balanced accuracy that beyond two standard deviations among the 10-fold cross-validation.

The proposed differential diagnosis model showed the best classification performance among all compared models, achieving a balanced accuracy of 79.7% for bvFTD, 81.9% for nfvPPA, 89.2% for svPPA, and an overall balanced accuracy of 83.6%. Table 2 shows the results of the ablation study to evaluate the performance of the proposed FTD subtype differential diagnosis model using 10-fold class-stratified nested cross validation, in terms of balanced accuracy for each subtype as well as the overall performance, and Figure 4 shows the corresponding box plot of the class-specific balanced accuracy as well as the overall multi-class balanced accuracy. When comparing single-type features as input, the thickness-only feature input (Table 2B; Figure 4 yellow) showed stronger discriminative power compared to the volume-only feature input (Table 2A; Figure 4 blue) for bvFTD, svPPA, as well as the overall performance. Interestingly, simply concatenating the volume and thickness feature types into a single input feature vector (Table 2C; Figure 4 green) resulted in reduced classification performance compared to the thickness-only feature input. On the contrary, the proposed multi-level parallel feature embedding approach (Table 2D; Figure 4 red) demonstrated performance improvement in terms of balanced accuracy for the classification of two out of the three FTD subtypes (bvFTD and nfvPPA), as well as the overall balanced accuracy.

**Table 2:** The ablation study of the FTD subtype differential model.

Feature Type	bvFTD	nfvPPA	svPPA	Overall
A) Volume	0.742	0.791	0.854	0.796
B) Thickness	0.781	0.790	0.885	0.819
C) Thickness + Volume	0.760	0.796	0.867	0.808
D) Thickness + Volume (multi-level)	**0.797**	**0.819**	**0.892**	**0.836**

The classification performances were reported as the mean balanced accuracy on the test sets across all the 10 outer folds of the nested cross-validation. Different combination of input features and network architecture design are reported, including: (1) volume-only input features; (2) thickness-only input features; (3) Joint volume and thickness input feature using single-level MLP; and (4) Joint volume and thickness input feature using two-level MLP. The balanced accuracy for each individual FTD subtype (bvFTD, nfvPPA, and svPPA) as well as overall balance accuracy was reported. Bold values indicate the model with the best performance in terms of mean balanced acuracy.

### 3.3 FTD subtype differential patterns through explainability deep learning

**Figure 5:**
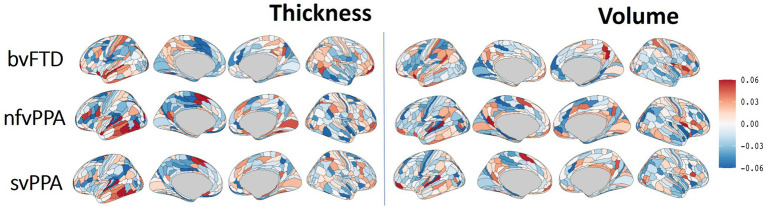
Differential cortical patterns for each of the FTD subtype. The cortical manifold plot visualizes populational average feature importance map using Integrated Gradient based feature importance analysis projected onto the template cortical manifold (HCP-MMP1 atlas) for both the cortical thickness (left) and volume (right) features. The color maps represent Integrated Gradient based feature importance scores ranging from −0.06 to 0.06. The magnitude of the feature attribution (i.e., absolute value) represent the influence of each feature towards the output classification, while the sign of the feature attribution (i.e., positive and negative) reflect the direction of the feature influence towards the classification output. Attributions that are close to zero represent features that have minimal influence in models prediction.

**Figure 6:**
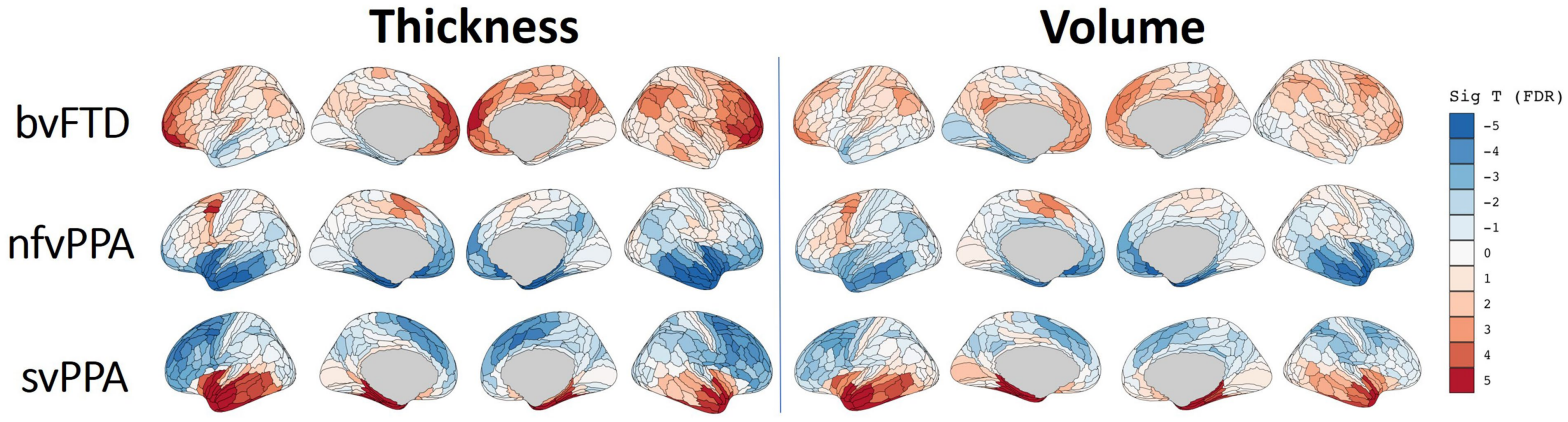
the statistical cortical mapping for each FTD subtype in which the patch-wise cortical features (both thickness and volume) were statistically compared with the combination of remaining populations that belong to the combination of the other two FTD subtypes.

The Integrated Gradient based FTD subtype feature attribute visualization patterns are shown in Figure 5 for both cortical thickness and volume features. The magnitude of the feature attributions (i.e., absolute value) represents the influence of each feature toward the output classification, while the sign of the feature attribution (i.e., positive and negative) reflect the direction of the feature influence toward the classification output. For example, for features with positive attributions (as shown in red), increasing in scalar value of the feature (i.e., structural volume or cortical thickness) will increase the likelihood of prediction for the correct FTD subtype; while for features with negative attribution (as shown in blue), decrease in scalar value of the feature will increase the likelihood of prediction for the correct FTD subtype. In other words, both the positive attributions (red) and negative attribution (blue) with the same Integrated Gradient value will have equivalent feature importance for making the correct classification, but with different direction of the influence. Attributions that are close to zero represent features that have minimal influence in the model prediction. Based on the thickness features (Figure 5, left), patches within the left temporal lobes appear to positively impact differentiation for both nfvPPA and svPPA. Regions from the inferior frontal and frontal operculum also positively influence the model for nfvPPA. For bvFTD, left-sided anterior temporal and frontal opercular/insular as well as bilateral frontal pole regions showed positive influences on the model, while cingulate and paracentral regions had negative influences (shown in blue). Volume-based features showed relatively diffuse differential patterns for both positive and negative influence than thickness features, although in generally similar overall patterns. This observation aligns with the results of the ablation study that thickness features showed stronger power to classify FTD subtypes compared to volume features. Figure 6 displays their corresponding patch-based statistical cortical mapping visualization, demonstrating canonical patterns of cortical atrophy in each subtype. Patterns of cortical atrophy in each subtype generally correspond to the Integrated Gradient based features of importance (i.e., in the temporal regions for svPPA and nfvPPA, and in frontal regions for bvFTD). However, it’s worth noting that the patterns of atrophy tend to be more evenly distributed across neighboring patches, whereas Integrated Gradient based feature importance displays a more scattered distribution.

## 4 Discussion

In this study, we developed a deep-learning-based framework for the identification and differentiation of three subtypes of FTD (bvFTD, nfvPPA, and svPPA) based on structural MRI data drawn from two multi-site neuroimaging consortiums. We showed that the ensembled DNN classifier achieved promising differentiation power, with a balanced accuracy of 0.80 for bvFTD, 0.82 for nfvPPA, and 0.89 for svPPA. We additionally implemented a novel feature visualization tool to identify the most discriminative cortical and subcortical regions and explore their clinical relevance, which can provide insights into the underlying neuropathological processes and aid in the development of targeted interventions for different FTD subtypes.

The high balanced accuracy achieved by the DNN classifier in this study is an important step towards developing more reliable tools for differentiating FTD subtypes using neuroimaging data. Machine learning methods have been extensively implemented in the differential diagnosis of Alzheimer’s disease (AD) from cognitively normal controls (Wang et al., 2007; Lucas et al., 2011; Raamana et al., 2014; Dominic et al., 2018; Popuri et al., 2018; Bae et al., 2019; Gyujoon et al., 2022), and between AD, FTD, and cognitively normal (CN) groups (Wang et al., 2016; Kim et al., 2019; Ma et al., 2020; Hu et al., 2021). Prior work has also implemented machine learning methods for differential diagnosis of PPA subtypes, including svPPA, nfvPPA and logopenic PPA (Agosta et al., 2015; Themistocleous et al., 2021). However, the performance of these models has been inconsistent, with few studies reporting high accuracy levels but with small sample sizes (McCarthy et al., 2018).

One of the challenges in machine learning classification of FTD subtypes is the significant clinical, pathological, and genetic heterogeneity of FTD, making it difficult to develop a universal model that can accurately classify all subtypes. Additionally, the lack of large and standardized datasets, as well as the variability in imaging protocols across studies, have also limited the generalizability. DNN classifiers have shown superior performance compared to traditional machine learning methods, such as support vector machines (SVM) and random forest for accurate classification of disease groups using neuroimaging data (Schmidhuber, 2015; Eslami and Saeed, 2019; Amini et al., 2021). On the other hand, end-to-end deep learning frameworks such as CNN-based usually require a large sample size for training. In the current study, we designed a multi-type feature extraction and multi-level feature embedding framework based on the multi-layer perceptron (MLP) framework, with dimension reduction and feature extraction achieved through neuroimaging-based preprocessing pipelines to extract the structural features from the raw T1 MR. Specifically, we demonstrated that the fusion of multi-type input features in DNN is most effective through multi-level parallel feature embedding, in which each feature type was embedded into independent feature-specific low-dimensional representation before fusion together for a higher-level concurrent representation learning. Our results (Table 2; Figure 4) demonstrated the effectiveness of such a multi-type feature fusion approach as compared to the naïve feature concatenation at the input layer. Such a multi-type parallel feature embedding framework could be generalizable to other multi-modal deep learning problems such as neuroimaging genomics (Mirabnahrazam et al., 2022).

Our results showed the highest balanced accuracy of classification for svPPA at 0.89. svPPA is commonly associated with striking asymmetric atrophy of the dominant hemisphere temporal pole (Rogalski et al., 2011). This distinctive atrophy pattern is usually due to the presence of TDP-43 Type C neuropathology in these regions (Kawles et al., 2022; Keszycki et al., 2022). The high discriminative accuracy found in the present study is, therefore, unsurprising given this distinctive neuropathological profile and resultant neuroanatomical pattern of atrophy. Regions of the temporal lobes were identified as most useful in the discrimination, both for nfvPPA and svPPA, potentially driven by the semantic and linguistic variations that are identified as clinical features to define these two FTD subtypes. Moreover, subcortical regions, including the hippocampus and amygdala, were identified by the feature visualization tool as aiding in the differentiation (Supplementary Figure S3), aligning with the fact that more posterior elements of the medial temporal lobe in svPPA spared (Tan et al., 2014).

For bvFTD, our classifier achieved a balanced accuracy of 0.80. Individuals diagnosed clinically with bvFTD typically show significant gray matter volume loss of the frontal and temporal lobes, with early and most significant loss of volume in the insula and anterior cingulate cortex (Seeley et al., 2008; Mandelli et al., 2016; Ranasinghe et al., 2016). The lower classification accuracy observed in bvFTD than in svPPA may represent the greater clinical, neuroanatomical, and pathological heterogeneity of bvFTD. Indeed, bvFTD can be due to underlying FTLD-Tau, FTLD-TDP, or less commonly, AD neuropathology (Peet et al., 2021). Based on the feature visualization map, brain regions that more strongly contributed to the classification of bvFTD vs. others include the left posterior insula, superior temporal gyrus, and right prefrontal lobe for cortical thickness. For volume-based input data, the right posterior cingulate and bilateral insular and frontal opercular regions were identified as strongly contributing to the classification. This is consistent with reports showing that atrophy of the insular cortex is common in bvFTD (Mandelli et al., 2016; Fathy et al., 2020) and has even been shown to correlate with key clinical features, such as social cognition (Baez et al., 2019).

Finally, we showed that nfvPPA classification balanced accuracy was 0.82. Patients who present clinically with nfvPPA typically show atrophy of the left inferior frontal, insular and premotor cortex (Agosta et al., 2015), consistent with the pattern of motor speech deficits that are observed clinically (Rogalski et al., 2011). The lower observed classification accuracy of bvFTD and nfvPPA may be attributable to overlapping neuropathological and neuroanatomical signatures, as both syndromes are frequently associated with FTLD-Tau pathology (Mesulam et al., 2008, 2014). In the feature visualization map, regions identified as contributing to the classification included the left lateral and medial temporal lobes, left inferior frontal lobe, and left paracentral/midcingulate for the thickness inputs. In addition, regions from the volume inputs that were identified as important included the left superior temporal and right frontal operculum. Interestingly, prior work by Mandelli et al. (2016) found that nfvPPA subjects showed greater atrophy in the left posterior insula, which corresponds more to speech production, whereas bvFTD subjects showed greater atrophy in the ventral anterior insula, which corresponds to social–emotional functions. We observed similar results in our feature importance map, with regions of importance for nfvPPA being more congruent with inferior frontal motor speech areas, while bvFTD areas of importance were more apparent in the posterior insula and the anterior superior temporal lobe. Feature visualization maps also indicated that the bilateral hippocampal and right amygdala volumes were important in the classification (Supplementary Figures). Analyses of subcortical structural changes in nfvPPA are limited. However previous research has indicated possible effects on structures of the basal ganglia due to their role in hypothesized speech production pathways (Mandelli et al., 2018).

### 4.1 Limitations and future directions

In the current study, we considered demographic information as cofounding factors and controlled their effects on neuroimaging features through a regression-based harmonization step (Ma et al., 2019). This harmonization approach has been shown to be effective in increasing the classification power when predicting the risk of future dementia onset (Popuri et al., 2020) and differentiating dementia subtypes (Ma et al., 2020).

Furthermore, disease subtypes might have populational prevalence among different demographic groups (Ma et al., 2022), and this information might aid discrimination. Indeed, incorporating demographic information into deep-learning frameworks has shown benefits to the deep-learning model in clinical applications such as dementia onset risk (Mirabnahrazam et al., 2022). Future directions of the current research could include investigating an alternative strategy to incorporate demographic information into the differential diagnosis framework instead of treating them as confounding factors in the harmonized preprocessing step, potentially improving the efficacy and generalizability of the differential diagnosis framework.

Additionally, our classification of interest was clinical diagnosis, as clinical syndromes are known to correspond more closely to neuroanatomical lesions as compared to neuropathology (Seeley et al., 2009). However, future research may choose to incorporate clinical, pathological, or genetic information to evaluate how this impacts classification accuracy.

In this work, we did not include cognitively normal control subjects as the healthy aging population due to insufficient samples, so we selected bvFTD as the reference group for data harmonization. Therefore, the resulting feature importance map mainly accounts for the more subtle differences among the three FTD subtypes rather than their differential atrophy patterns compared to the cognitively normal subjects. Future studies may choose to incorporate a large representative healthy aging population to be regarded as the reference group to achieve the most unbiased data harmonization (Ma et al., 2020), as well as extend to multi-syndrome dementia subtypes (Lampe et al., 2022) to capture brain patterns that include both predominant pathological factors as well as secondary subtype-driven differential patterns that are more likely to be subtle and relatively more heterogeneous.

We used structural features from T1-weighted MRI in the current study to derive differential features for detecting subtypes within FTD. Extension of current work could involve additional neuroimaging modalities such as diffusion tensor imaging (DTI) (Torso et al., 2020) or functional MRI (fMRI) (Gonzalez-Gomez et al., 2023). Another future direction for dealing with limited features would be to use a self-supervised approach as a feature extractor, to be trained on larger datasets, to extract disease-agnostic generalized neuroimaging features in lower dimensions, and then train a using the low-dimension representation space (Krishnan et al., 2022; Tang et al., 2022; Huang et al., 2023).

Finally, in terms of the model explainability, we mainly focused on using the deep-learning-based integrated gradient to derive the feature importance map. In follow-up studies, other feature importance methods, especially model-agnostic approaches such as SHAP (SHapley Additive exPlanations) (Lundberg and Lee, 2017) and multi-type feature permutation tests (Mirabnahrazam et al., 2022) could be incorporated to achieve more comprehensive and comparative analysis on the clinical explainability of deep-learning-based models.

## 5 Conclusion

In conclusion, we present here what we believe represents the first study to use a deep neural network classifier to differentiate the FTD subtypes of bvFTD, nfvPPA, and svPPA with feature visualization. We showed promising differentiation power using a combination of feature harmonization and a parallel multi-type feature embedding framework. Our approach has several potential clinical applications. For example, it could be used to identify at-risk populations for early and precise diagnosis, leading to more effective intervention planning. Further, our work may also help to advance our understanding of the underlying neurobiological mechanisms of FTD, providing important insights into the pathophysiology of the disorder.

## Data availability statement

The original contributions presented in the study are included in the article/Supplementary material, further inquiries can be directed to the corresponding authors.

## Ethics statement

Ethical approval was not required for the study involving humans in accordance with the local legislation and institutional requirements. Written informed consent to participate in this study was not required from the participants or the participants’ legal guardians/next of kin in accordance with the national legislation and the institutional requirements.

## Author contributions

DM: Conceptualization, Data curation, Formal analysis, Funding acquisition, Investigation, Methodology, Project administration, Software, Validation, Visualization, Writing – original draft, Writing – review & editing. JS: Conceptualization, Data curation, Formal analysis, Funding acquisition, Investigation, Methodology, Resources, Software, Validation, Visualization, Writing – original draft, Writing – review & editing. HR: Conceptualization, Data curation, Investigation, Project administration, Resources, Supervision, Validation, Writing – review & editing. KK: Data curation, Validation, Methodology, Writing – review & editing. SL: Investigation, Methodology, Resources, Validation, Writing – review & editing. JB: Investigation, Supervision, Validation, Writing – review & editing. SC: Funding acquisition, Investigation, Supervision, Validation, Writing – review & editing. MG: Investigation, Validation, Writing – review & editing. KP: Investigation, Methodology, Writing – review & editing. MB: Investigation, Methodology, Supervision, Writing – review & editing. LW: Conceptualization, Funding acquisition, Investigation, Methodology, Project administration, Resources, Supervision, Writing – review & editing.

## Footnotes

^1^ https://www.allftd.org/

^2^ https://memory.ucsf.edu/research-trials/research/allftd

^3^ http://4rtni-ftldni.ini.usc.edu/
