# Supplementary figures and images for: Differential diagnosis of frontotemporal dementia subtypes with explainable deep learning on structural MRI
Source: Front Neurosci. 2024 Feb 7;18:1331677. doi: 10.3389/fnins.2024.1331677 (PMC10879283; doi:10.3389/fnins.2024.1331677)

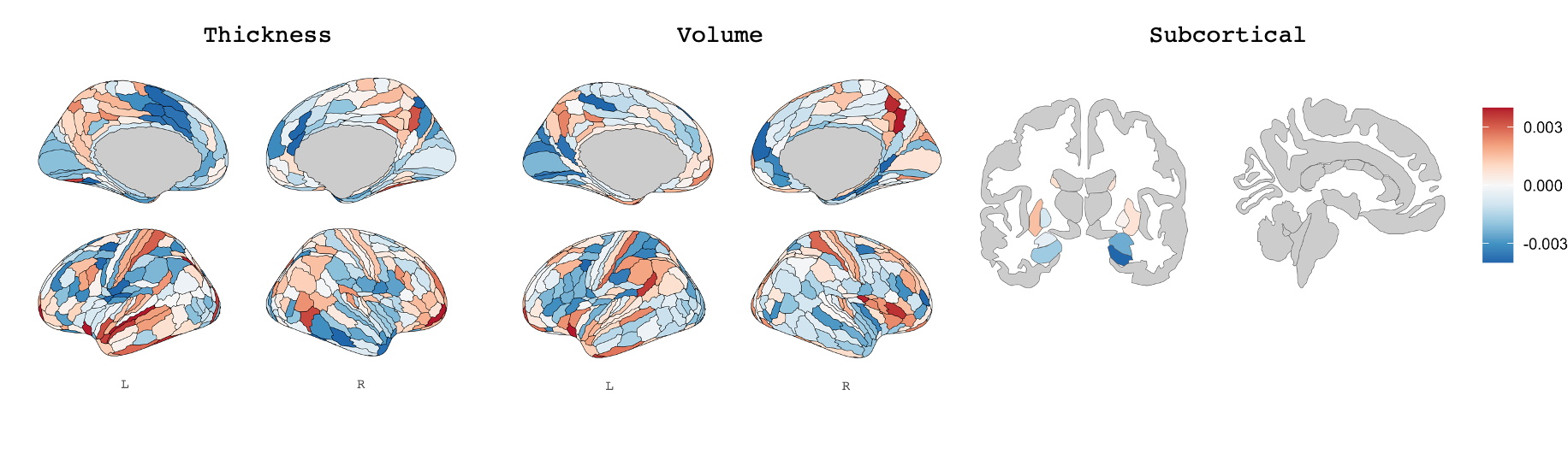

Supplement: Supplementary file 1 [file Image_1.jpg]

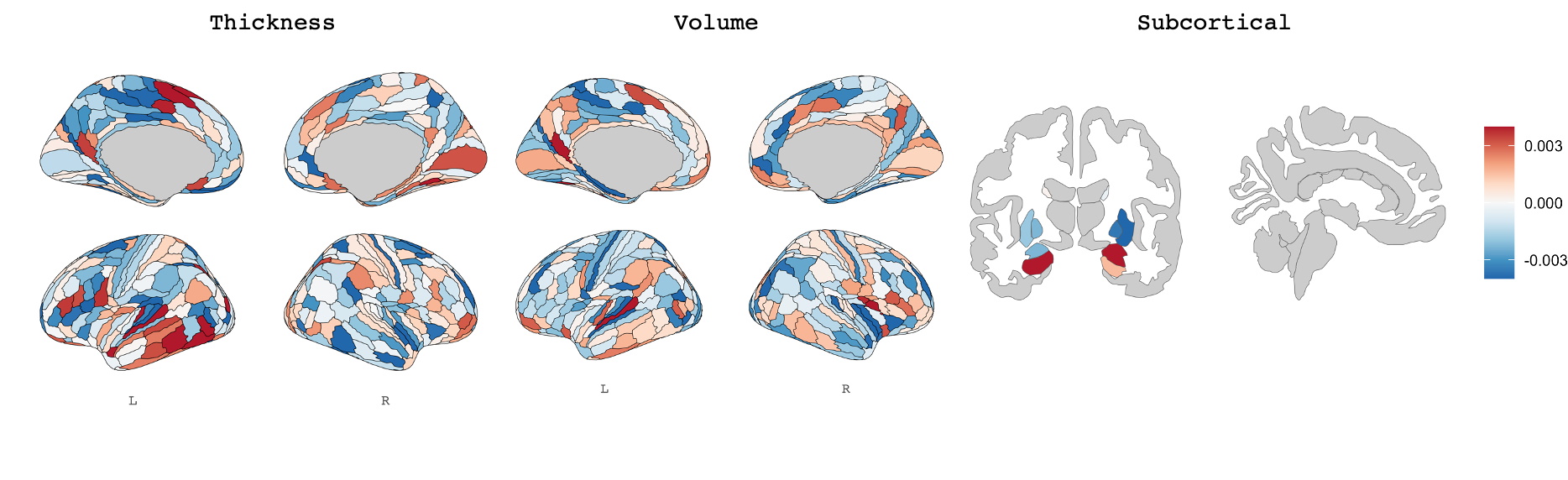

Supplement: Supplementary file 2 [file Image_2.jpg]

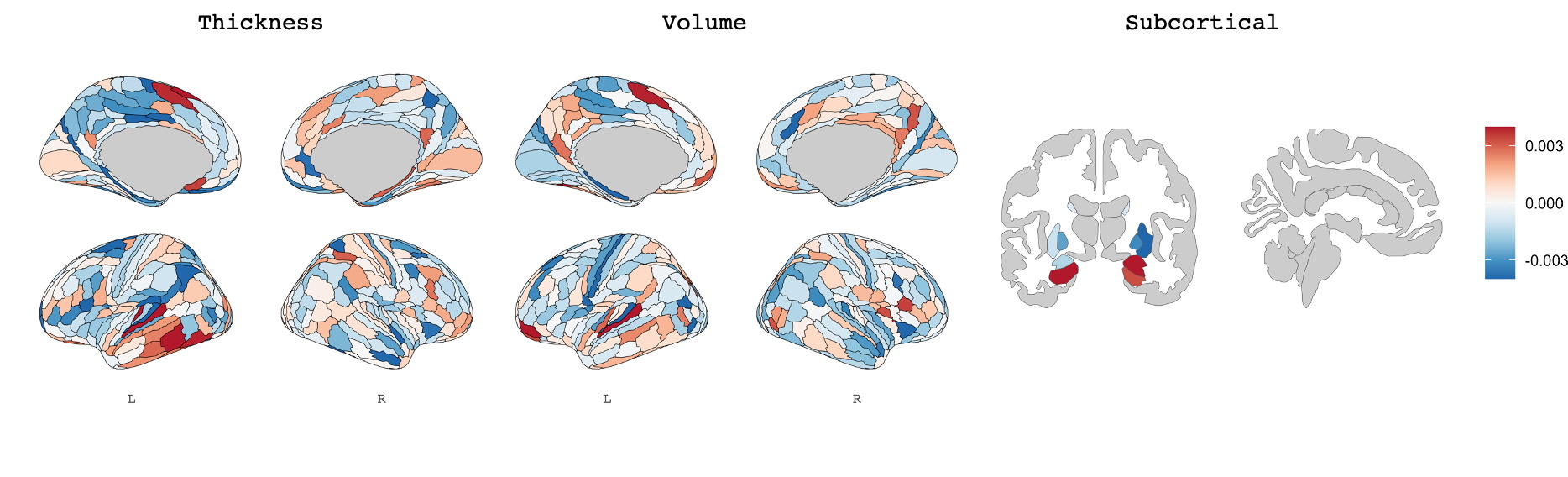

Supplement: Supplementary file 3 [file Image_3.jpg]
